# Effects of Autonomy Support and Emotion Regulation on Teacher Burnout in the Era of the COVID-19 Pandemic

**DOI:** 10.3389/fpsyg.2022.846290

**Published:** 2022-04-25

**Authors:** Mei-Lin Chang, Rachel E. Gaines, Kristen C. Mosley

**Affiliations:** ^1^Department of Secondary and Middle Grades Education, Kennesaw State University, Kennesaw, GA, United States; ^2^Department of Educational Psychology, University of Texas at Austin, Austin, TX, United States

**Keywords:** emotion regulation, COVID-19 pandemic, teacher burnout, work autonomy support, cognitive reappraisal

## Abstract

The COVID-19 pandemic has exacerbated levels of stress and anxiety for P-12 teachers around the globe. The present study aims to understand teachers’ emotional experiences and feelings of burnout during the pandemic, and how individual (i.e., emotion regulation strategies) or contextual factors (e.g., school administrative support) intersect with different facets of their emotional experiences. Using a sequential explanatory mixed methods design, we collected and examined survey and interview data from teachers in the southeastern United States. The structural equation model confirmed the relationships among the following latent variables: negative emotion, emotion regulation, autonomy support, burnout, and teacher enthusiasm. Qualitative findings provide further insight in the contextualized nature of these relationships and how they play out across various schools and districts.

## Introduction

Compared with other professionals, teachers in P-12 schools experience higher levels of stress (Ferguson et al., 2012; Landsbergis et al., 2020), and the COVID-19 pandemic exacerbated levels of stress and anxiety for teachers around the globe (Kim et al., 2021; Pressley, 2021). The workload and emotional labor required of teachers were also significant prior to the pandemic (Chang, 2009), but have only increased as teachers have encountered unprecedented challenges including abrupt transitions between virtual, hybrid, and in-person instruction in socially distanced classrooms. To ensure continuity, teachers rapidly adapted curricula and instruction to fit new modalities, occupational responsibilities, and classroom environments. The additional burdens and stress of “pandemic teaching” have been detrimental to teachers’ mental health and, consequently, increased burnout (Klapproth et al., 2020; Sokal et al., 2020; Jakubowski and Sitko-Dominik, 2021).

Previous research has found that teachers use a variety of strategies to regulate their emotions while teaching, including cognitive reappraisals, which have been identified as protective against burnout (Sutton et al., 2009; Yin et al., 2016; Taxer and Gross, 2018; Chang and Taxer, 2021). Yet, despite the unequivocal international consensus that teachers’ social-emotional well-being and mental health were negatively impacted by the pandemic, little is known about how they regulate the emotions involved in pandemic teaching. The purpose of this research is to understand teachers’ emotional experiences and feelings of burnout during the pandemic, and how individual (i.e., emotion regulation strategies) and contextual factors (e.g., school administrative support) intersect with different facets of their emotional experiences.

### Teacher Stress and Burnout in the Pandemic

COVID-19 and the requisite adjustments implemented to mitigate its spread have drastically changed students’ and teachers’ lives. School closures and at-home quarantines, the most widely used measures at the beginning of the pandemic (Esposito and Principi, 2020), affected 63 million teachers in 165 countries. Globally, 1.3 billion learners participated in alternative school experiences, such as virtual and hybrid learning models (Joshi et al., 2020). The widespread implementation of such measures had a deleterious effect on teachers’, students’, and parents’ mental health (Ahmed et al., 2020; Marshall et al., 2020; Ozamiz-Etxebarria et al., 2021). Teachers, among others, have experienced decreased mental health and well-being due to the isolating and challenging nature of remote and virtual work (Kaden, 2020; Marshall et al., 2020; Pressley, 2021). Specifically, moderate to severe levels of anxiety, depression, and stress during this pandemic have become commonplace for many teachers (Klapproth et al., 2020; MacIntyre et al., 2020; Jakubowski and Sitko-Dominik, 2021).

According to Kim and Asbury (2020), teaching became more cognitively and emotionally taxing during the pandemic due to the challenges of teaching from home. For example, teachers experienced a lack of support in transitioning classroom teaching to online platforms, external distraction and family interruption throughout the academic day, and logistical barriers to conducting proper and valid assessments (Joshi et al., 2020). Several institutional hurdles prevented teachers from effectively transforming teaching for virtual instruction, such as insufficient technical infrastructure and support, limited training in online teaching or software, and inadequate communication from administration (Joshi et al., 2020). Furthermore, the unexpected and extended time spent teaching virtually necessitated an increase in parent communication, thus creating additional demands on teachers (Wu et al., 2020). Many teachers had their own children learning at home as well, which required them to juggle their teaching and parenting roles (Pressley, 2021). Teachers participating in prior research noted the pandemic felt “like a rug had been pulled [from under them]” (Kim and Asbury, 2020, p. 1070), and navigating the changes made “[their] brain feel like a browser with 100 tabs open” (Kim et al., 2021, p. 309).

As the pandemic shifted from wave to wave, schools in some areas reopened for in-person learning at various points between 2020 and 2021. When these schools reopened, many teachers were forced to adapt their instruction to a socially distanced version of the face-to-face classroom. Still others were required to use a synchronous hybrid model in which virtual and in-person students were instructed simultaneously. Recent research has found teachers experience more significant and intense negative emotions and lower job satisfaction (Lindner et al., 2021) in these instructional transitions, and consistently report medium-to-high levels of anxiety, depression, stress and burnout (Jakubowski and Sitko-Dominik, 2021).

### Emotion Regulation in Curbing Burnout

Emotion is an integral part of teaching and learning (Hargreaves, 1998). It has interpersonal and intrapersonal facets which are often influenced by social, cultural, and political contexts (Liljestrom et al., 2007; Chang, 2009; Fried et al., 2015; Gaines et al., 2019). Because of the emotional labor required in teaching, emotional exhaustion has been identified as a salient factor of teacher burnout, and the connections among emotion, emotion regulation, and burnout have been explored in several studies (Sutton, 2004, 2007; Chang, 2009, 2013; Jennings and Greenberg, 2009; Tsouloupas et al., 2010; Keller et al., 2014). In examining ways to curb teacher burnout, researchers have identified emotion regulation and coping as effective means to reduce the emotional exhaustion associated with burnout (Brackett et al., 2010; Chang, 2013; Durr et al., 2014).

Gross (1998) defines emotion regulation as “the processes by which individuals influence which emotions they have, when they have them, and how they experience and express these emotions” (p. 275). Gross also suggests there are generally two forms of emotion regulation: cognitive reappraisal and expressive suppression. Through cognitive reappraisal, an individual changes their thinking about a situation to decrease its emotional impact (Lazarus and Alfert, 1964). Expressive suppression occurs when an individual inhibits ongoing, emotionally expressive behavior. Previous research suggests these specific emotion regulation strategies may be adaptive or maladaptive depending on the context (Grandey and Melloy, 2017; Taxer and Gross, 2018).

Prior research has examined outcomes related to teachers’ use of these emotion regulation strategies. For example, teachers’ use of cognitive reappraisals has been shown to mediate the relationship between emotional job demands and teacher well-being (Tsouloupas et al., 2010; Yin et al., 2016; Chang and Taxer, 2021). That is, when teachers reported engaging in cognitive reappraisal, they reported experiencing less emotional exhaustion, despite their jobs being emotionally demanding (Chang, 2013; Chang and Taxer, 2021).

Conversely, suppression has little impact on unpleasant emotions while also consuming cognitive resources which, in turn, constricts the capacity to absorb information and access memory throughout the duration of the emotion regulation period (Gross, 2002). Suppressing emotions, also referred to as surface acting, has therefore been found to deplete teachers’ cognitive resources, which may lead to impaired instruction and decreased well-being (Carson, 2007; Chang, 2013; Burić and Frenzel, 2020) as well as greater burnout and lower job satisfaction (Keller et al., 2014).

### Teacher Enthusiasm

Teacher enthusiasm is an affective component which “reflects the degree of enjoyment, excitement, and pleasure that teachers typically experience in their professional activities” (Kunter et al., 2008, p. 470). Keller et al. (2016) further define it as the conjoined occurrence of positive affective experience, that is teaching-related enjoyment, and the behavioral expression of these experiences. Teacher enthusiasm has been connected with supportive classroom environments (Turner et al., 2002), quality of teaching (Feldman, 2007), and positive student outcomes (Frenzel et al., 2009). It is also positively correlated with teachers’ overall job satisfaction, and lower levels of emotional exhaustion (Kunter et al., 2008, 2011, 2013). In addition, Cobb and Foeller (1992) also found positive links between teacher enthusiasm and autonomy.

### Teachers’ Job Autonomy During the Pandemic

Job autonomy is considered a major factor in an individual’s assertion of whether their job is exhausting or satisfying (Wharton, 1993; Moller et al., 2006), and recent research has demonstrated the benefits of autonomy-supportive leadership in promoting worker empowerment and self-initiation (Ryan and Deci, 2017). Autonomy-supportive leaders aim to understand the needs of each individual and encourage input and involvement. In examining factors that may lessen teacher stress and burnout, Collie (2021) collected data between March and May 2020 from 325 Australian teachers and found that autonomy-supportive leadership was associated with greater buoyancy and, in turn, lower somatic burden, stress related to change, and emotional exhaustion. In contrast, autonomy-thwarting or controlling practices involve pressuring individuals to feel, act, and think in particular ways, and have been positively associated with emotional exhaustion (Ryan and Deci, 2017).

Research on P-12 teachers has consistently identified positive outcomes associated with increased teacher autonomy. For example, a study of 251 South African teachers found increased job autonomy to be related to teachers’ increased feelings of meaningfulness and engagement in their work (Peral and Geldenhuys, 2016). Similarly, teacher autonomy may be a viable means of increasing their well-being (Collie et al., 2018), as previous studies have reported decreased feelings of autonomy to be a common experience in the teaching profession (Greenville-Cleave and Boniwell, 2012).

### Present Study

Due to the pivotal role of teachers’ emotional experience in outcomes such as stress and burnout, and the exacerbation of these outcomes during the COVID-19 pandemic, the present study aims to understand teachers’ emotional experiences and feelings of burnout during the pandemic and how individual (i.e., emotion regulation strategies) or contextual factors (e.g., school administrative support) intersect with various facets of their emotional experiences.

The overarching research question guiding this study was, *during pandemic teaching, what impact did contextual factors (e.g., confirmed COVID cases in schools, policy changes) and teachers’ emotion regulation strategies have on teachers’ emotional experiences and feelings of burnout?* Derived from the literature, we hypothesized the following relationships in explaining teachers’ emotional experiences and feelings of burnout in the pandemic: (1) teachers’ negative emotions would covary with the number of confirmed cases in the school; (2) emotion regulation through cognitive reappraisal would buffer the impact of teachers’ negative emotions on overall feelings of burnout; (3) emotional regulation through expression suppression would increase feelings of burnout; and (4) teachers’ perceived autonomy support (PAS) and enthusiasm would serve as protective factors from feelings of burnout. After examining these hypotheses in Phase 1, we further explored how these relationships manifest in Phase 2 through interviews with teachers about their individual experiences across various school contexts.

## Materials and Methods

A sequential explanatory mixed methods design (Creswell and Plano Clark, 2011) was employed to understand teachers’ emotional experiences during the COVID-19 pandemic. The first phase involved a quantitative study in which we examined the effects of emotion regulation and PAS on teacher burnout. The second phase involved a qualitative study in which we further explored teachers’ experiences teaching virtually, hybrid, and in-person during the pandemic; their emotional experiences and regulation strategies; and their intentions to stay/leave the field. The method is considered *sequential* and *explanatory* because the initial quantitative results are explained further through the subsequent qualitative inquiry. The explanatory sequential design represents a strategy to ensure the reliability of the quantitative portion of the study, as well as the dependability of the qualitative portion, thereby supporting the overall trustworthiness of the study overall.

Specifically, in Phase 1, survey data was used to test a theoretical model aimed at understanding the relationships between teachers’ emotional experiences (i.e., negative emotions and enthusiasm), emotion regulation strategies, PAS, and burnout during the pandemic. In Phase 2, six one-on-one interviews with teachers were analyzed to further understand how teachers used emotion regulation strategies to manage the emotional labor of pandemic teaching, and how their emotional experiences during the pandemic varied according to school and personal contexts.

### Sample

Participants in Phase 1 were 284 full-time teachers from the southeastern United States. Teachers were recruited from May to August 2021 through local teacher organizations, professional learning communities, and graduate teacher education programs. Participant survey links were shared *via* teacher organizations’ social media and graduate teacher education program course announcements. Teachers were informed that clicking the survey meant they consented to participate in the study. The online survey was submitted by 342 teachers, but only 284 surveys were complete and included for analysis. Although this creates potential for self-selection bias (e.g., some participants may have been too stressed to complete the survey), the 83% completion rate is greater than the average expected completion rate for an online survey containing more than 61 items, on which fewer than 80% of responses are typically completed (Liu and Wornski, 2018).

Participants in Phase 1 included teachers who taught in elementary schools (48.2%), middle schools (24.4%), and high schools (27.3%). We elected to sample from all levels K-12 to increase the representativeness and size of our sample. Approximately 34% of teachers (*n* = 110) taught in Title-I schools (lower socioeconomic status). In terms of teaching modality and COVID-related protocols during the span of August 2020 to May 2021, only 8% of all participants reported they taught only in the format of in-person teaching, and 92% of them had engaged in some form of hybrid instruction: approximately 53.2% taught in hybrid formats in which more class time was spent on in-person than virtual instruction, 23.8% spent equal amounts of class time on in-person and virtual instruction, and 23.0% spent more class time on virtual than in-person instruction. Nearly half of the participants (48.9%) reported the number of confirmed COVID-19 cases in their own schools was greater than 30, and more than half (61.4%) reported their schools were never closed (i.e., no virtual or in-person instruction) due to the pandemic.

Participants in Phase 2 were recruited from a follow-up survey in Phase 1. Phase 1 participants were invited to indicate their willingness to be interviewed for Phase 2. The 85 participants (29.9%) who volunteered to be interviewed were prompted to share the name of the school district in which they worked. With our interest in the influence of district-level decisions about how schools would operate during the pandemic, participants in Phase 2 were purposely selected from schools in different districts in the metro-city area. Knowing we would have a relatively small sample for the qualitative phase, it was important to select participants who varied in ways that directly addressed our research questions and represented diverse perspectives while maintaining commonality in areas that would allow us to make reasonable comparisons between them. See Table 1 for additional demographics for Phase 2 participants.

**TABLE 1:** Demographic and school characteristics of Phase 2 participants.

Participants (County)	School context and subject	Personal demographic info
Barbara (Dayflower County)	Private middle school, Science	European American, career changer, veteran teachers, curriculum director, started graduate school fall of 2020 when school re-opened.
Dara (Camellia County)	Public high school, English	African American, 15 years of teaching. Leadership changed (superintendent switched) during summer of 2020
Irene (Dogwood County)	Public high school, Mathematics	European American, 12 years of teaching. 6 years of middle school, and 6 years of high school
Kristi (Jessamine County)	Public high school, Economics	Hispanic American, career changer, worked in business as a Chief Financial Officer before teaching
Monica (Wisteria County)	Public middle school, Science	African American, 14 years of teaching. Science Team Lead.
Nancy (Spotting County)	Public high school, English	European American, novice teacher in the 4^th^ year of teaching.

*Participant and county names are pseudonyms. All participants were female.*

### Instruments

In addition to demographic items, the Phase 1 survey was composed of items asking teachers about the negative emotions and enthusiasm (i.e., enjoyment of and excitement about teaching) they experienced at work during the pandemic, their PAS, and two additional measures: Emotion Regulation (Gross and John, 2003) and Maslach Burnout Inventory-Educator Survey (Schaufeli and Salanova, 2007), each of which is described next.

#### Reliability and Construct Validity of the Scales in Phase 1

##### Teacher Emotions

A total of eight items were included to measure teachers’ emotional experiences of teaching during the pandemic. To capture the negative emotions teachers felt most often at work during the pandemic, we included a list of five discrete negative emotions (i.e., anxiety, anger, frustration, sadness, stress). Participants rated the frequency of the negative emotions they experienced on a scale of 1–6 (1 = Never, 2 = A few times a semester, 3 = A few times a month, 4 = Once a week, 5 = A few times a week, and 6 = Almost daily).

To capture teachers’ positive emotions during the pandemic, we adopted three-items from the enthusiasm scale (Kunter et al., 2008, 2011) to measure teachers’ positive emotional experiences while teaching during the pandemic. The items included, “I enjoy teaching my class(es),” and “I find the subject(s) I teach exciting and try to convey my enthusiasm to the students.”

Confirmatory factor analysis (CFA) was conducted using these eight items, and the fit indices indicated a good fit (χ^2^ = 26.10, *df* = 16, RMSEA = 0.04, SRMR = 0.06, GFI = 0.98, and CFI = 0.99). Cronbach’s alphas were also sufficient for the current study: 0.87 for the *negative emotion* subscale and 0.86 for *enthusiasm* subscale.

##### Emotion Regulation

An eight-item emotion regulation scale was adapted from Gross and John (2003) to capture teachers’ patterns of emotion regulation in the classroom during the pandemic. Four items were used to capture reappraisal strategies, and four were used to capture suppression strategies. Using a 6-point Likert-type scale, participants rated their level of agreement (1 = Very strongly disagree, 6 = Very strongly agree) with statements about their emotion regulation strategies (e.g., “When I want to feel less of an unpleasant emotion, I change what I’m thinking about”; “I control my emotions by not expressing them”). CFA was conducted using these eight items, and the fit indices indicated a good fit (χ^2^ = 50.61, *df* = 19, RMSEA = 0.07, SRMR = 0.09, GFI = 0.96, and CFI = 0.96). The Cronbach’s alphas in the present study were 0.86 for the *reappraisal* subscale and 0.75 for the *suppression* subscale.

##### Modified Maslach Burnout Inventory-Educator Scale

Teacher burnout was measured by the modified Maslach Burnout Inventory-Educator Scale (MBI-ES; Schaufeli and Salanova, 2007). The measure is composed of seven items spanning three dimensions: emotional exhaustion (e.g., “I felt emotionally drained by my work”), depersonalization (e.g., “I worry that this job is hardening me emotionally”), and inefficacy (e.g., “I can’t solve the problems that arise in my teaching like I used to”). Participants were asked to report the frequencies of their burnout symptoms on a scale from 1 to 6 (1 = Never, 2 = A few times a semester, 3 = A few times a month, 4 = Once a week, 5 = A few times a week, and 6 = Almost daily). High scores on the items indicate higher frequencies of burnout symptoms. CFA was conducted using these seven items, and the fit indices indicated a good fit (χ^2^ = 32.48, df = 11, RMSEA = 0.08, SRMR = 0.06, CFI = 0.98, and GFI = 0.97). The Cronbach’s alpha of the overall burnout scale is 0.85.

##### Perceived Autonomy Support

The research team developed six items to measure teachers’ perceptions of autonomy support while teaching during the pandemic. Participants were shown items such as “School administrators considered my personal preferences when determining the modality of my classes,” and “School administrators provided sufficient support for modifications I had to make to my teaching,” to which they responded using a 6-point Likert-type scale (1 = Very strongly disagree, 6 = Very strongly agree). CFA was conducted using these six items, and the fit indices indicated a good fit (χ^2^ = 19.08, df = 7, RMSEA = 0.07, SRMR = 0.06, CFI = 0.96, and GFI = 0.98). The Cronbach’s alpha of the PAS scale is 0.70.

#### Interview Protocols and Procedures in Phase 2

To understand how school and individual context influence teachers’ emotional experiences during pandemic teaching, a semi-structured interview protocol was developed to include the following themes: background questions (e.g., years of teaching); experiences teaching virtually, hybrid, and in-person; emotional experiences and emotion regulation strategies; attrition/retention intentions; and resources and perceived support related to coping with pandemic teaching. An initial draft of the protocol was created by one member of the research team and shared with two other members, who provided feedback on the wording and order of questions as well as questions to be added or removed. A revised version of the protocol was then shared with the same two team members, who recommended further revisions and approved the finalized protocol.

The team member who had created the original draft of the interview protocol conducted all the interviews *via* Microsoft Teams video conferencing platform and recorded the audio of each interview. Once all interviews were complete, recordings were uploaded to Otter.ai for transcription. Members of the research team reviewed the transcriptions and audio recordings and corrected transcriptions as needed.

### Data Analysis

#### Analysis of Phase 1 Survey Data

Basic statistical analyses were conducted using SPSS 28.0. Two main statistical procedures, CFA and structural equation modeling (SEM) were conducted using LISREL version 11 (Jöreskog and Sörbom, 2021). CFA was conducted to confirm the factor structures of the latent variables in the model and reported in the previous section.

To determine the extent to which the proposed theoretical model was supported by the collected sample data, SEM was used to test the fit of the model. The benefit of using SEM to test model fit is its ability to simultaneously adjust for measurement error in both dependent and independent variables (Schreiber et al., 2006; Schumacker and Lomax, 2010). A covariance matrix was generated to test the model using the maximum likelihood method of estimation.

LISREL provides fit indices to judge the goodness of fit between the empirical data and the model-implied data structures. Goodness of fit was assessed using the chi-square goodness of fit (χ^2^), the root mean square error approximation (RMSEA), and the comparative fit index (CFI). The sample size of the present study is considered large (*n* = 284, >200). Thus, RMSEA and CFI were chosen because these two indices are less sensitive to sample size than others (Fan et al., 1999). Model fit is considered excellent when the CFI is greater than 0.95 and acceptable when the CFI is no less than 0.90. In addition, RMSEA must be less than 0.06 and 0.08 for an excellent model fit, and 0.08 and 0.10 for an acceptable fit (Schreiber et al., 2006).

Estimation of direct and indirect effects were tested within LISREL. Specifically, the indirect paths from negative emotions to burnout and enthusiasm through the mediators were estimated in addition to the hypothesized mediation model (Preacher and Hayes, 2008).

#### Data Analysis for Phase 2 Interview Data

Transcripts from the six semi-structured interviews served as primary qualitative data sources. Transcripts were analyzed using a multi-phase inductive approach based on open, axial, and selective coding as well as constant comparison (Glaser, 1978; Strauss and Corbin, 1990). First, three members of the research team independently read the same two transcripts and, based on the research question guiding the study, created tentative (open) codes and made memos about potential categories. After comparing coding, they established an initial set of six categories and nearly 40 working codes.

Next, the same three individuals independently read an additional transcript and attempted to apply the preliminary codes, while noting potential revisions that may improve or clarify codes/categories. Once again, the three met to compare coding, consolidate and reorganize codes (i.e., axial coding), and revise the list as needed. The resulting code book contained six broad categories and 45 specific codes. It should be noted that 14 of these codes referred to specific emotions teachers expressed in their interviews. We believed it was valuable to capture their emotions as precisely as possible while coding, particularly with the knowledge that these codes would likely be consolidated in the context of the quantitative findings; however, we could not disaggregate the specific emotions if we coded more broadly (i.e., negative and positive emotions). When it was unclear from the transcript alone what specific emotion was being expressed, we revisited audio recordings and reached decisions based on tonal cues.

Next, the remaining six members of the research team were trained on the code book and each transcript was re-coded independently by two members of the research team. The coding for each transcript were sent to an auditor, who sent questions/comments back to each coder. Coders responded to the auditor’s feedback and revised their coding, at which point the auditor calculated percent agreement (min = 74%, mean = 80%).

The finalized codes were applied to the transcripts in Atlas.ti 9.1.3, at which point the specific emotion codes were consolidated into two broader codes (i.e., negative emotions and positive emotions) and one code that appeared only once across the data set was excluded (Hill et al., 1997). Table 2 includes selected codes and categories from the resulting code book that are most relevant to the current study.

**TABLE 2:** Qualitative code book.

Category	Code	Example
**Autonomy, Control, and Voice**: Areas in which teachers were explicitly granted or denied autonomy or control.	Curriculum and Instruction	“I don’t know how exactly we’re going to do the digital class I have this school year…They said it was totally left up to the teacher…” (Irene).
	Platform/LMS /tech	“Some people still use Google Meet though, because they were used to Google Meets, so some teachers are kind of doing what they want to do. But we’re supposed to be using Zoom.” (Dara)
	Schedule/time	“When [school leaders] were like, ‘Let’s have a one-hour meeting about self-care,’ and people were like, ‘I think it would be better self-care for me to use that time grading.”’ (Nancy).
	Modality	“No one ever asked us. Wisteria never sent a survey… I would have been so much less stressed if they would have just let the teachers who are high risk or who didn’t want to come back do the virtual piece, and let the teachers who were willing to come in do the face to face.” (Monica)
	Lack of interest in teacher input/expertise	“And it was not protesting bringing the students back. It was protesting the way that the county had decided to reopen. And they did it without input from teachers. And they did it without consulting what the day to day would look like from us.” (Kristi)
**Emotion Regulation and Coping:** Strategies and resources that supported participants’ ability to mitigate demands and regulate their emotions	Intentions to leave/stay	“There’s certainly just general aspects of being in the classroom that were wearing me down that I knew I wanted to move out of the classroom. So, maybe I felt a little stronger this past year, perhaps.” (Barbara)
	Reappraisal	“[As] bad as it was, having to work from home for those few months in the spring of 2020, I think it gave me some more perspective for working with those virtual kids for the whole of last year.” (Nancy)
	Suppression	“[It] was so much with we’re going back here, we’re not going back…We just were always on edge, so we’re just gonna do what they tell us to do and not think about anything else. Because if you keep thinking about what they may do, it increases the anxiety.” (Dara)
	Wellbeing promoting practices	“I think meditation, I started yoga, and that was definitely helpful. Driving with no sound on the way home.” (Monica)
	School-based resources	(a) Collegial support (“Then we started pulling together our own sort of in-house team to get ready and I was part of that. And coming to that meeting made me feel a little better,” Barbara) (b) Administrative support (“I think my principal was more accommodating as a human to the teachers during the pandemic. A lot of the pressure that other schools or other teachers may have felt, my principal was more lax on the teachers because people were worried, people were stressed out, people had family members die,” Dara) (c) Technical support (“Having that teacher that was like a liaison or [virtual course] expert, he was constantly coming out with videos, you need to know how to do this, here’s a video for you. So he was always available and he tried to stay ahead of the curve,” Irene) (d) Social-emotional resources (“To my principal’s credit…Wednesdays, she would have a social worker come in and go through mindfulness exercises and things like that,” Monica)
**Perception of Safety:** Aspects of participants’ teaching context that informed their perceptions of safety at school.	Lack of concern for teachers’ safety	“[The former superintendent] did an interview and the interviewers were asking him to address the teachers who are concerned about COVID, and he said something like, ‘Oh, the teachers are just confused.”’ (Nancy)
	Inadequate protocols, policies, and guidance from leadership	“We really didn’t have any guidance as to what to tell parents. We didn’t have a closure matrix. We didn’t have a set number of cases. They did not contact trace.” (Monica).
	Lack of emotional safety	“I attempted to express my frustrations, but then I was told I was negative and not a team player, which was, in essence, ‘We don’t care,’ you know?” (Kristi)
	Strong, strictly implemented protocols and procedures	“We all stayed healthy, if anything healthier than other years because I didn’t get a cold. You know, all the things we get from the kids we didn’t get…because we had masks on.” (Barbara)
	Lack of transparency about risk	“[Cases] were not reported. Either students and their parents were not going to the hospital or a doctor, and if they were, we do not feel like they were reporting back because we had some student that were not there for a long time, but in their computer system, it wasn’t mark ‘COVID excused absence.”’ (Irene).
**Emotions:** The specific emotions participants attributed to their pandemic teaching experiences	Negative emotions	Anger/frustration, Anxiety, Discomfort/unease, Exhaustion/overwhelmed, Fear, Hopelessness/loss of excitement, Isolation/loneliness, Sadness/despair/grief, Stress, Uncertainty
	Positive emotions	Gratitude/enjoyment, Optimism/excitement, Pride

*Additional codes and categories were developed before quantitative data had been analyzed. This table includes only those codes and categories that were relevant in explaining the statistically significant relationships identified in the quantitative phase.*

## Results and Findings

### Results of Phase 1

#### Preliminary Results

The hypothesized full measurement model includes subscales measuring six latent variables including negative emotions, emotion regulation strategies (cognitive reappraisal and suppression), PAS, burnout, and enthusiasm. Table 3 shows the means, standard deviations, and bivariate correlations for the latent variables. Teachers reported very high levels of negative emotions (*M* = 4.95, *SD* = 1.04) and feelings of burnout (*M* = 4.22, *SD* = 1.18). In terms of emotion regulation strategies, they reported higher use of suppression (*M* = 5.10, *SD* = 1.28) than cognitive reappraisals (*M* = 4.47, *SD* = 1.12). They also reported very low levels of PAS (*M* = 2.20, *SD* = 1.01). There are significant correlations among most variables. These correlations followed the definitions of the variables, providing preliminary evidence for the construct validity of the scales.

**TABLE 3:** Zero-order correlations of latent variables in the model.

	1	2	3	4	5	6
(1) Negative Emotion						
(2) Expressive Suppression	0.14					
(3) Cognitive Reappraisal	–0.26**	0.12				
(4) Perceived Autonomy Support	–0.49**	–0.15*	0.24**			
(5) Burnout	0.72**	0.15*	–0.29**	–0.41**		
(6) Enthusiasm	–0.32**	–0.07	0.34**	0.30**	–0.46**	
Means	4.95	5.10	4.47	2.20	4.22	4.89
*SD*	1.04	1.28	1.12	1.01	1.18	1.14
Cronbach’s alpha	0.87	0.73	0.82	0.70	0.85	0.86

**Correlation is significant at the 0.01 level (two-tailed).

*Correlation is significant at the 0.05 level (two-tailed).

#### Structural Equation Modeling Results

**FIGURE 1:**
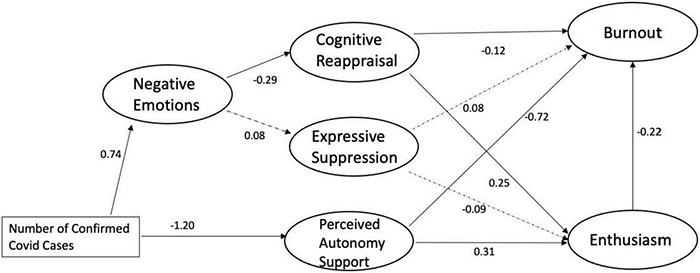
Structural equation model for the relationship between confirmed COVID-19 case numbers, negative emotion, emotion regulation, perceived autonomy support, burnout, and enthusiasm (standardized coefficients). All coefficients are significant (*p* < 0.05) except the paths with dotted lines.

Before submitting the theoretical model for testing, the full measurement model was tested and was found to approach a good fit based on the fit indices (χ^2^ = 661.17, *df* = 350, χ^2^/*df* = 1.88, RMSEA = 0.05, CFI = 0.92). Once the construct validity of the measurement model was established, the structural model was tested to examine the direct and indirect relationships between negative emotions, cognitive reappraisal, suppression, teacher PAS, burnout, and enthusiasm. The fit indices indicated an acceptable fit for the model overall (χ^2^ = 783.77, *df* = 382, χ^2^*/df* = 2.05, RMSEA = 0.06, and CFI = 0.90, see Figure 1).

All hypotheses were confirmed by the SEM results except the relationships between suppression with other variables: (1) as predicted in our hypothesis, number of confirmed COVID-19 cases reported at school was a significant path leading to teachers’ negative emotions during the pandemic (β = 0.74, *p* < 0.05). In addition, the number of confirmed COVID-19 cases reported at school also negatively covaried with teacher PAS (β = –1.20, *p* < 0.05). (2) When examining the mediating effects of teachers’ emotion regulation strategy use, teachers’ negative emotions significantly covaried with cognitive reappraisal (β = –0.31, *p* < 0.05) but not with suppression (β = 0.09, *p* > 0.05). Further, as hypothesized, cognitive reappraisals negatively covaried with burnout (β = –0.12, *p* < 0.05), and positively covaried with enthusiasm (β = 0.25, *p* < 0.05). (3) Unexpectedly, suppression did not significantly covary with either burnout (β = 0.08, *p* > 0.05) or enthusiasm (β = –0.09, *p* > 0.05); (4) however, as expected from our hypotheses, teacher PAS and enthusiasm were confirmed as protective factors against burnout. Specifically, autonomy support negatively covaried with burnout (β = –0.72, *p* < 0.05), and positively covaried with enthusiasm (β = 0.31, *p* < 0.05). Furthermore, teacher enthusiasm negatively covaried with burnout (β = –0.22, *p* < 0.05).

#### Indirect Effects of Negative Emotions

Direct and indirect effects among the latent variables were estimated in LISREL. In the model, there are two significant indirect effect paths. Both paths are derived from negative emotions, one leading to burnout (*z* = 0.07, *p* < 0.05), and the other to enthusiasm (*z* = –0.09, *p* < 0.05).

#### Discussion of Structural Equation Modeling Results

Our Phase 1 study further extends our understanding of teacher emotional experiences during the pandemic caused by COVID-19. The SEM model testing allowed us to examine several emotion-related variables simultaneously and understand how these variables intersect with each other. Beyond confirming several hypotheses in the literature, our contribution is unique as the study examined the constructs related to teacher emotions in the context of pandemic teaching, and it identified the positive and potential protecting effects of cognitive reappraisals, PAS, and enthusiasm against feelings of burnout.

Aligned with the literature regarding teacher emotional experiences in the pandemic teaching, teachers in our study reported very high levels of negative emotions and feelings of burnout (Jakubowski and Sitko-Dominik, 2021; Lindner et al., 2021). Our SEM model further identified a direct link between the number of confirmed COVID-19 cases in school and teachers’ negative emotions. In addition, with increased numbers of confirmed COVID-19 cases reported at school, teachers were more likely to report a lack of autonomy support. This finding is aligned with the common theme reported by other studies in which teachers felt unsupported in the situations when they were forced to adjust and transform their traditional in-person teaching to virtual, hybrid, or socially distanced classrooms (Joshi et al., 2020; Kim and Asbury, 2020).

Further, the SEM results indicate the positive effects of cognitive reappraisals in curbing feelings of burnout (Chang, 2013; Yin et al., 2016; Chang and Taxer, 2021) and promoting teacher enthusiasm. When adopting cognitive reappraisal strategies, teachers examined stressful pandemic events from different perspectives, thus avoiding being trapped in negative emotions and maintaining their enthusiasm toward teaching. This result also mirrors recent findings regarding cognitive reappraisal as protective factors from psychological distress among teachers (Jennings et al., 2017) or from stress and anxiety symptoms in COVID-19 isolated participants (Xu et al., 2020). Although the link between cognitive reappraisal and enthusiasm has not been verified in prior literature, our findings corroborate Moè and Katz’s (2021) conclusions in viewing reappraisal as a key factor for teachers to adopt more positive and motivating teaching styles.

The strength of PAS appeared to be the most significant path in the model that negatively covaried with burnout. This highlights the importance of soliciting teacher inputs and providing flexibilities and instrumental support for teachers to adapt to the challenges of pandemic teaching (Collie, 2021).

Unexpectedly, our SEM findings did not support the linkage between expressive suppression and teachers’ overall feelings of burnout (Carson, 2007; Keller et al., 2014; Burić and Frenzel, 2020). A possible explanation is that the context of pandemic teaching may have changed the strength of the effects of this maladaptive way of emotion regulation. Although depleting, it is not a major contributor to teachers’ overall feelings of burnout. This warranted further investigation of our Phase 2 study to understand the discrepancy of our findings with previous literature.

### Phase 2 Explanatory Findings

Qualitative coding was conducted without knowledge of the quantitative results to mitigate potential confirmation bias. However, in accordance with explanatory mixed-methods research, the quantitative results offered necessary context for *interpreting* the qualitative analyses (Mason, 2006). The following sections offer evidence from the qualitative data that enriches the quantitative results by providing context for and nuances of the relationships found to be significant in the model.

#### COVID Case Counts, Perceived Autonomy Support, and Negative Emotions

In the Phase 1 study, number of confirmed COVID-19 cases at school was negatively associated with PAS and positively associated with negative emotions. To advance our understanding of these relationships, we examined qualitative data pertaining to teachers’ perceptions of safety (as a proxy for COVID-19 case counts) and how they related to teachers’ perceived autonomy and emotions.

The most frequently endorsed code regarding perception of safety addressed school/district leadership’s lack of concern for teachers’ health and safety. Understandably, teachers expressed negative emotions including (but not limited to) anger, anxiety, and stress in these instances. For many participants, school/district leaders demonstrated their lack of concern through opacity about transmission and case counts. Monica explained,

We didn’t have a set number of cases. They did not contact trace…I didn’t find out that kids in my classroom had COVID unless a parent told me. And when I went to my administration and said, “Well, how are you contact tracing if you didn’t tell me that the student had COVID?” [They responded,] “Well, we looked at your seating charts.” Well, kids don’t stay in their seats, so that isn’t [going to work].

She referred to this experience as “the beginning of the height of my anxiety, because it just felt like I was on my own as far as, are they even going to tell me [if I was exposed to COVID]?” In such circumstances, teachers like Monica were confident that the number of *confirmed* COVID cases drastically underestimated the actual numbers of cases in their schools, forcing them to guess what the actual number of cases may be. This may explain in part why the reported number of COVID cases would be associated with both negative emotions and perceived lack of autonomy support.

Kristi reported similar behavior from her school/district leadership. In fact, when a group of teachers in her school walked out during their lunch break (and returned after lunch to fulfill their teaching duties) in protest “of the fact that the safety protocols were not being appropriately communicated [and] what was being communicated to parents was not actually happening in the day-to-day school operations,” the faculty “received a letter from our superintendent telling us that the teachers who walked out were selfish.” Although Kristi did not explicitly state that she was angry about this incident, her tone of voice in the audio recording clearly expressed anger and frustration.

These cases overlap with PAS interestingly as well, as both involve teachers’ attempts to advocate for themselves and their students by voicing concerns about the lack of transparency from administration, and the potential repercussions for teachers’ and students’ health. Yet, in both cases, teachers’ concerns were disregarded or met with censure. Even Irene, who expressed the most positive emotions and enthusiasm of any participant, reported experiencing negative emotions because, in her school,

…cases were not reported. Either students…were not going to the hospital or a doctor, [or] if they were, we did not feel like they were reporting back [that they had COVID], because we had some students that were not there for a long time. But in the computer system, it wasn’t marked “COVID excused absence.” So we’re like, where is this person? And they come back [and say], “Oh I was sick.” And we can’t ask them, so that avoided a lot of people getting in quarantine because they wouldn’t tell us.

Once again, when teachers expressed concern and frustration over the lack of transparency and unsafe teaching conditions, school leaders initially made minimal effort to respond. Irene recalled a beginning-of-the-year math department meeting attended by the principal in which “several teachers got very upset that they could not see family members because they had to come back to work and they didn’t know if they were exposed or not…” When teachers asked the principal why they were not being informed of confirmed cases, the principal claimed, “They could not release that to us because of…HIPPA.” Unlike Monica and Kristi’s schools though, Irene noted, “Eventually they did start notifying teachers [if] there was a case in your class…That took a little while, and I think that was because *so many people were upset*” (emphasis added). This may explain in part the strong negative association between the number of confirmed COVID cases and PAS: teachers who felt informed about accurate COVID case counts/exposures and had a voice in designing and implementing safety protocols not only felt safer at school, but actually influenced policies in ways that may have improved health outcomes for educators and learners.

#### Cognitive Reappraisal, Emotion, and Burnout

Although relationships among cognitive reappraisal, emotions, and burnout are well-established in the literature (Tsouloupas et al., 2010; Yin et al., 2016; Chang and Taxer, 2021), our qualitative data illustrate how these relationships played out in schools during the pandemic. Specifically, the model suggests that teachers who experienced more negative emotions were less inclined to engage in cognitive reappraisals, which were predictive of positive emotions and protective against burnout. Examining teachers’ pre-pandemic and pandemic-specific negative emotions and the nature of their reappraisals may help explain why reappraisal was more effective for some than others.

Apart from Barbara (who had already left the classroom at the time of her interview), all participants used reappraisal to help regulate their emotions. The nature of these reappraisals, however, differed noticeably between Monica, who intended to leave teaching, and the other four participants, who either plan to stay or expressed uncertainty about their intentions (see Table 4). As evidenced in Table 5, Irene, Dara, Nancy, and Kristi described reappraisals that supported their emotion regulation in occupationally sustaining ways that both decreased negative emotions and increased positive emotions in line with the quantitative findings.

**TABLE 4:** Effect of pandemic on intentions to stay or leave.

Participant	Prior concerns	Effect of COVID on intentions	Intentions	Actions planned/Taken
Irene	None stated	None stated	Stay	N/A
Dara	None stated	“I talked about it.”	Stay	“I haven’t really done anything activity to exit.”
Nancy	None stated	“…maybe casually considered it a couple more times [during the pandemic].”	Stay	“I need to see if I’m still enjoying it once we…go back to normal.”
Kristi	Lack of respect for teachers	“What are we doing [to actually serve students during the pandemic]?”	Conflicted	“And that’s where I was like, is it my time to leave? I don’t want it to be that, because I’m a teacher who cares deeply about her subject.”
Barbara	“There’s certainly general aspects of being in the classroom that were wearing me down [before the pandemic].”	“…so maybe that felt a little stronger this past year.”	Transition to Admin	Took position in administration
Monica	Long-standing concerns about district-level leadership	“[That’s] one thing I’m not willing to give up is my physical safety for this profession…because again, they haven’t told us how many kids are coming back…we don’t know anything.”	Leave	“Definitely looking to leave within hopefully the next two years if not sooner…my husband and I already talked about that and my family is in support…so we definitely have an exit plan.”

**TABLE 5:** Reappraisal in support of emotional regulation and teacher sustainability.

Participant	Initial appraisal	Reappraisal	Emotion regulation
Irene	Expressed frustration about teachers’ lack of instructional autonomy and increased workload due to the mandated use of [virtual course].	“[Second] half of the year, things changed because I knew how to manipulate stuff [in virtual course]. *I knew what mistakes I had made first semester. I was like, Oh, I need to not do that second semester. So second semester got a lot better*” (emphasis added).	Decreased frustration; increased excitement
Dara	“[My] mind [was] spiraling all these insane possibilities…”	“[Therapy] helped me to be more positive and realize that some of my thoughts are irrational…It’s definitely helped me cope during this time period.”	Increased coping, decreased anxiety and fear.
Nancy	“It was rough…having to work from home for those few months in spring 2020…Because I was not really doing great when I had to work from home.”	“And I think as bad as it was…I think it gave me some more perspective for working with those virtual kids [in hybrid classes] last year. [Anytime] I was working and talking to those students, I just remember how hard it is to be isolated and try to go through school, which is supposed to be an interactive, collaborative place.”	Decreased frustration, increased compassion and sense of connection to students.
Kristi	Expressed anger and frustration about school and district mandates that removed teacher autonomy over grades, student attendance, etc.	“I think my building principal did the best she could with the resources she had, but she is held accountable to the county administrator, so she was stuck in the middle…” “At the end…the positive side of COVID was that we did form a tight knit bond in that maybe this person might not have asked for help in the past, but because it was a COVID year, we were just all hands on deck…”	Decrease anger/frustration at principal; increased empathy/compassion. Increased connection and support among colleagues

For Monica, reappraisal did support coping and emotion regulation. For example, before COVID-19, Monica described herself as “the teacher who worked every weekend, all the time, I wanted everything to be perfect,” but she recognized that this was negatively affecting her mental health and she began limiting the amount of time she spent working when she was at home with family. However, during the pandemic, her negative feelings about school/district leaders and public education more generally, and her desire to leave the field within the next few years yielded reappraisals that further distanced her from students and her work. This type of distancing is indicative of depersonalization, and therefore increased feelings of burnout. Specifically, out of frustration about the way she and other teachers were treated throughout her pandemic teaching experiences, and with the knowledge that she would not be in the classroom for much longer anyway, she decided to be more selective about what she was willing to do at work. For instance, when told she had to cover absent teachers’ classes without receiving sub pay, “I told them up front that I refuse to sub for anyone…I didn’t care if they dropped my pay or took my time. I did not care because I refuse.” Ultimately, unlike the other participants, Monica’s reappraisals appeared to redirect her emotional labor (i.e., anger, frustration, stress) without truly reframing her experiences in ways that reduced the exhaustion that comes from such negative emotional experiences.

#### Protective Potential of Perceived Autonomy Support

The relationship between PAS, burnout, and various measures of teacher wellbeing are well-established in the literature, such that the associations between PAS, positive emotion, and burnout do not require extensive explanation. However, we present Irene as an illustrative example of how PAS promoted positive emotions and teacher sustainability in the context of pandemic teaching.

Although no participants *explicitly* attributed positive emotions to PAS, Irene did perceive autonomy support across all four of areas for which we coded (curriculum and instruction, modality, platform/LMS/technology, scheduling) and expressed optimism and excitement more frequently than other participants. Irene’s district was unique in offering teachers the option of teaching face-to-face or virtually. Irene recalled, “I believe the email said if you would like to teach an online class when the school reopened in fall 2021, and I volunteered…I think it was all volunteer.” Although Irene did not ascribe a specific positive emotion to this experience, she “was surprised that they considered teacher input,” which conveys a pleasant emotional response. She also recalled that when it came to returning to school, “there wasn’t too much pushback” from teachers, once again suggesting that teachers had fewer negative emotions, if not more positive emotions. This seems to bear out as well in Irene’s description of the emotions she experienced most often during pandemic teaching: “I would say stress, just because of completely changing your teaching, delivery, and learning a new system [but] I wouldn’t say I was any more sad or more happy. That was about the same.” Irene also expressed optimism and excitement about her ongoing and future teaching despite the continued uncertainty about the pandemic, largely because of the autonomy she felt as an instructor. She spoke of how, once given the freedom to teach her course as she saw fit, she was optimistic about her ability to improve the quality of virtual instruction (“It will be more rigorous. I think it will go smoothly because I’ll keep working on it until it does go smoothly”). Moreover, Irene repeatedly noted that she is sustained and excited by her work when it poses new challenges (“Even if it was the same topic, it was a different way of doing it and I did enjoy that part”; “…I’m teaching statistics for one class this year, and so that challenge is also sustaining me because I’ve never taught it before”), such that the pandemic itself was personally motivating.

## Discussion of Results and Findings

Results of the SEM and the qualitative findings fairly mirror the larger body of literature on the relationships among the latent variables we included in the model: negative emotion, emotion regulation, autonomy support, burnout, and teacher enthusiasm. The present study extends the literature by exploring these relationships in the context of pandemic teaching using a sequential explanatory mixed-methods approach, which strengthens our ability to contextualize these relationships on a deeper level.

### Negative Emotion and Burnout Intensified Due to COVID-19

Teachers in our sample experienced high levels of stress, negative emotions (e.g., anger, frustration, anxiety), and burnout in the context of pandemic teaching, all of which align with recent studies in Austria, Britain, Canada, Chile, Germany, the Philippines, and Poland (Klapproth et al., 2020; MacIntyre et al., 2020; Jakubowski and Sitko-Dominik, 2021; Lindner et al., 2021; Oducado et al., 2021). The concern for safety appears to ground these negative emotional experiences, as evidenced by our quantitative and qualitative findings. Personal safety concerns were particularly associated with the reported number of confirmed COVID cases at school, which were also associated with teachers’ negative emotions. The qualitative data support these results as evidenced by teachers’ perceived lack of safety due to inconsistency in contact-tracing policies and lack of transparency regarding confirmed COVID cases in several schools. Teachers reported high levels of anger and frustration regarding these lax protocols.

### Emotion Regulation as Mediators to Curb Burnout and Sustain Teacher Enthusiasm

Consistent with literature regarding the benefits of cognitive reappraisals (Tsouloupas et al., 2010; Chang, 2013; Yin et al., 2016; Chang and Taxer, 2021), our findings suggest that emotion regulation through cognitive reappraisal allowed teachers to mediate the negative influence of intensified negative emotions on burnout. As suggested by Lazarus and Alfert (1964), through cognitive reappraisals, one changes their thinking about a situation to decrease its emotional impact. Although pandemic teaching is demanding and can be emotionally taxing, teachers who engaged in cognitive reappraisal also reported experiencing lower levels of burnout in both phases of the study. Furthermore, these teachers reported higher levels of enjoyment and enthusiasm toward teaching, mirroring prior findings (Yin et al., 2016; Chang and Taxer, 2021).

Evidence from the qualitative data elucidate the constructive processes of reappraisals and how they decreased the intensity of negative emotions while sustaining teachers’ commitment to and enthusiasm in teaching. One participant, Nancy, explained how reflecting on the negative emotions she had experienced while teaching in isolation at home made her more mindful and empathetic about virtual students’ social-emotional needs. Such empathy was particularly impactful when her school initiated simultaneous hybrid instruction. Ultimately, she came to value her own experiences of isolation and loneliness during the first phase of lockdown in the pandemic as a means to form stronger connections with her virtual students. Her evolving perspectives regarding her own feelings of disconnection while quarantined offer a compelling example of how cognitive reappraisal can support coping by promoting positive emotions and reminding teachers of what they find sustaining in their work.

Another contextualized process of cognitive reappraisal that emerged from the qualitative data involves participants’ recognition of how adapting instruction for a virtual environment had forced them to develop new skills and resources that will help them serve future students. In line with the findings from Bubb and Jones (2020), we found that learning to use new technology/platforms and creating additional instructional resources were demanding and exhausting processes, yet teachers generally recognized how developing online resources would have long-term benefits and improve the quality of their instruction.

Surprisingly, use of suppression as a means of emotion regulation to mediate the effects of negative emotions with burnout was not significant in our SEM model. Although the literature suggests that suppression consumes and depletes teachers’ cognitive resources, which leads to impaired instruction and decreased well-being (Carson, 2007; Chang, 2013, 2020; Burić and Frenzel, 2020) as well as greater burnout and lower job satisfaction (Keller et al., 2014), our quantitative findings did not confirm this relationship. As such, we did not report on the qualitative findings pertaining to expressive suppression in our results.

However, it is noteworthy that our qualitative findings, unlike the quantitative results, are consistent with work from MacIntyre et al. (2020) and suggest that certain teachers masked or avoided talking about their own negative emotions with students during the pandemic, which may have contributed to their burnout. Taxer and Gross (2018) suggest that individuals’ goals for regulating their emotions could be multifaceted (e.g., self- and/or student-focused), and teachers often regulate their emotions according to either instrumental (e.g., improve teaching effectiveness) or hedonic (i.e., ensure certain emotions are not felt) goals, both of which are evidenced in interview participants’ rationales for expressing or hiding their negative emotions in front of students. Specifically, those who had instrumental goals for expressing their own emotions believed that doing so improved their teaching effectiveness, while also supporting their emotion regulation. This is exemplified by Nancy, the novice high school teacher who chose to express her negative emotions to better connect with students and demonstrate that her classroom was a safe place to be vulnerable and take risks, which is often associated with improved academic outcomes (e.g., Sharma, 2015; Deveci and Aydin, 2018). Teachers with student-focused or *extrinsic* hedonic goals tend to “focus on emotionally supporting their students by ensuring that the students were experiencing positive emotions or not experiencing negative emotions” (Taxer and Gross, 2018, p. 183). This is exemplified by veteran teachers Kristi and Monica, who chose to perform positivity and enthusiasm to protect what they perceived to be emotionally vulnerable students. In the limited sample of the present study, our findings seemed to suggest hedonic goals may have contributed to these two teachers’ burnout and intentions to leave the field. This mirrors Burić and Frenzel’s (2020) suggestion that teachers, often out of dedication to their students, take on additional emotional labor (e.g., suppression) despite the personal psychological cost (Wang et al., 2019).

Implications for such findings include the need to consider teachers’ emotions in their work and how they can be supported in healthfully coping with the difficulties of the job–both during and irrespective of the pandemic. School leadership teams should consider how teachers can be supported not just for their instructional needs, but for their emotional and coping-related needs, too. Examples could include providing workshops for mindfulness practices in cultivating teachers’ emotional awareness and promoting cognitive reappraisals for positive well-being (Keng et al., 2016; Jennings et al., 2017) or offering on-site opportunities for the incorporation of teacher affinity and/or support groups. Although these resources do not address the root systematic and contextual factors that lead to high rates of stress and burnout in teaching, they do help teachers develop wellbeing promoting skills and practices that will serve them across contexts and domains.

### Lack of Autonomy Support and Teacher Burnout

Besides the intensified negative emotions, the confirmed case numbers at schools also contributed to lower PAS which in turn led to teacher burnout. Although our data were collected in one state in the U.S., district-level leaders in the state wield broad control over district schools. Therefore, we purposefully selected interviewees to represent school districts that varied in setting and implementing protocols for pandemic teaching. This element of diversity provided additional insights into the tremendous impact of district- and school-level leadership on teachers’ emotional experiences of pandemic teaching.

Discrepancies among districts regarding COVID-related policies were apparent among the interviewees, including mask requirements, ratio of in-person and remote instructional days, solicitation of input from teachers regarding preferred modality, contact-tracing protocols, and overall transparency regarding confirmed cases. Results from the quantitative and qualitative phases align with previous findings that autonomy-supportive leadership during the pandemic was associated with greater buoyancy and, in turn, lower somatic burden, stress related to change, and emotional exhaustion (Joshi et al., 2020; Collie, 2021).

As evidenced in the qualitative data, as well as numerous recent studies, the additional workload involved in adapting instruction to new modalities contributed to teachers’ stress and negative emotions in pandemic teaching (Alea et al., 2020; Fauzi and Khusuma, 2020). A lack of sufficient resources and autonomy support from school and district leaders intensified negative emotions and stress, which consequently exacerbated teachers’ feeling of burnout and threatened their well-being. This finding is consistent with several existing studies which also identified lack of administrative support or work autonomy as contributive to teacher burnout and stress during the pandemic (Joshi et al., 2020; Sokal et al., 2020; Zhou and Yao, 2020; Pressley, 2021). Teachers were deprived of autonomy, control, and voice when leadership teams established policies and protocols for reopening schools without considering teachers’ perspectives, or when confirmed case counts were not shared with teachers or parents. Although school and district leaders were forced at times to make abrupt, top–down decisions based on the rapidly evolving nature of COVID-19, our study speaks to the potential long-term implications of the hyper-centralization that often comes with a crisis. Specifically, those teachers who expressed intent to leave also attributed their attrition to the lack of respect and trust between administrators and teachers that was fomented or exacerbated during the pandemic.

The emotional labor of pandemic teaching is enormous, and several teachers reported the need to seek professional mental health support. The destructive consequences during pandemic teaching have also affected their engagement and commitment to teaching. In discussing the impact of leadership style and work engagement, Deci et al. (2017) suggested autonomy support as one of the core elements explaining work commitment. Autonomy support is also considered as one of the basic psychological needs to promote well-being and better work outcomes. To promote teachers’ well-being and combat stress in the context of pandemic teaching (or post-pandemic teaching), school leadership teams should, as often as possible, consider more autonomy-supportive practice, such as acknowledging teachers’ perspectives, offering choices, including them in decision making, and providing meaningful feedback to prompt intrinsic motivation and engagement and to limit psychosomatic symptoms, emotional exhaustion, and turnover.

## Conclusion, Limitations, And Implications

As we conclude this study, COVID-19 continues to shape teaching and learning globally. Despite a burgeoning body of literature on COVID-related teacher stress, anxiety, and depression, there is limited research available on teachers’ emotions, emotion regulation, and coping. The current study elucidates protective factors to ease burnout and sustain teachers’ enthusiasm even in this unprecedented moment. Moreover, most existing research on pandemic teaching has been either quantitative or qualitative. Thus, our sequential explanatory mixed methods design is a noteworthy contribution to the literature on teacher emotions during the pandemic.

As with all research, our study has limitations. Specifically, the generalizability may be limited based on our sampling. We relied on snowball sampling given the demanding nature of pandemic teaching. The sample was collected from one state in the U.S, which had notably more confirmed cases than other states at the onset of data collection (1 million + confirmed cases during 2020–2021). Additionally, we collected data several months after schools first closed for the pandemic, such that teachers were asked to recall their experiences during the first year of pandemic teaching. Therefore, as with all *post hoc* research, data may be impacted by memory bias. Finally, the generalizability of the qualitative findings could have been improved by including elementary educators in the sample, which consisted exclusively of secondary teachers. Future research should include longitudinal data to advance our understanding of the long-term impact of pandemic teaching experiences on teachers’ emotional well-being, emotion regulation, and longevity.

Two important implications can be derived from this study. First, as previously noted, to support teachers’ well-being and mitigate stress in the context of pandemic teaching (and post-pandemic teaching), school leadership teams should consider implementing autonomy-supportive practices to greatest extent possible (and practical). Our qualitative findings demonstrate that leaders can support teachers’ sense of autonomy through small acts such as soliciting and acknowledging teachers’ perspectives. These are other autonomy-supportive practices, such as offering choices related to their work requirements and providing meaningful feedback can prompt teachers’ intrinsic motivation and engagement while limiting psychosomatic symptoms, emotional exhaustion, and turnover. Second, this study provides evidence for the emotional work in which teachers engage and the negative toll it can take on their well-being. School leadership teams should consider whether they are presently supporting teachers in this aspect of their work and, if not, how they can better address teachers emotional and coping-related needs just as they typically address their instructional needs. For example, offering non-mandated professional development related to cognitive reappraisal, providing teachers opportunities to develop and partake in teacher affinity and/or support groups, and giving teachers’ access to psychosocial and emotional support *via* school counselors or clinical social worker embedded in schools may help teachers manage the unique, emotional demands of their work.

## Data Availability Statement

The raw data supporting the conclusions of this article will be made available by the authors, without undue reservation.

## Ethics Statement

The studies involving human participants were reviewed and approved by Institution Review Board of Kennesaw State University. The patients/participants provided their written informed consent to participate in this study.

## Author Contributions

M-LC and REG designed the mixed-methods study together. M-LC collected the survey and interview data, and analyzed the quantitative data. REG led the research team to develop the code book for the interview data, and analyzed the qualitative data along with KCM. All authors contributed to the write-up of the manuscript.

## Conflict of Interest

The authors declare that the research was conducted in the absence of any commercial or financial relationships that could be construed as a potential conflict of interest.

## Publisher’s Note

All claims expressed in this article are solely those of the authors and do not necessarily represent those of their affiliated organizations, or those of the publisher, the editors and the reviewers. Any product that may be evaluated in this article, or claim that may be made by its manufacturer, is not guaranteed or endorsed by the publisher.
